# Effects of Different Draw Solutions on Biogas Slurry Concentration in Forward Osmosis Membrane: Performance and Membrane Fouling

**DOI:** 10.3390/membranes12050476

**Published:** 2022-04-28

**Authors:** Yun Li, Xiaomin Xie, Rongxiu Yin, Qingzhao Dong, Quanquan Wei, Bangxi Zhang

**Affiliations:** 1 College of Resource and Environment, Qingdao Agricultural University, Qingdao 266109, China; liyun2015@cau.edu.cn (Y.L.); dong1024@stu.qau.edu.cn (Q.D.); 2 School of Environmental Science and Engineering, Shandong University, Qingdao 266237, China; xiexiaomin@sdu.edu.cn; 3 Tea Research Institute, Guizhou Academy of Agricultural Sciences, Guiyang 550006, China; yrxzhr@126.com; 4 Institute of Agricultural Resources and Environment, Guizhou Academy of Agricultural Sciences, Guiyang 550006, China; weiquan0725@163.com

**Keywords:** forward osmosis membrane, biogas slurry, draw solution, membrane fouling

## Abstract

Biogas slurry poses a severe challenge to the sustainable management of livestock farms. The technology of the forward osmosis (FO) membrane has a good application prospect in the field of biogas slurry concentration. Further research is needed to verify the effects of different draw solutions on FO membranes in biogas slurry treatment and the related membrane fouling characteristics. In this study, three different draw solutions were selected to evaluate the performance of FO membranes for biogas slurry concentration. Membrane fouling was investigated by characterization after FO membrane treatment to identify fouling contaminants. The result showed that FO membrane treatment can realize the concentration of biogas slurry and MgCl_2_ as the draw solution has the best effect on the concentration of biogas slurry. The different draw solutions all contributed to the efficient retention of most organics and TP while each treatment was ineffective at retaining nitrogen. The cake layer that appeared after the biogas slurry was concentrated covered the surface of the FO membrane. Some functional groups were detected on the surface after membrane fouling, such as C–O and C=C. Moreover, the C element accounts for 57% of the main components of the cake layer after the membrane fouling. Membrane fouling is caused by both organic fouling and inorganic fouling, of which organic fouling is the main reason. This study provides a technical reference for the high-value utilization of biogas slurry.

## 1. Introduction

Traditional energy sources such as coal and oil are increasingly depleted and accompanied by pollution. Some new energy sources are gradually being widely used, such as biofuels [1]. Therefore, using biogas projects to develop biomass energy and adjust the energy structure has become a necessary approach [2]. Biogas projects use wastes to produce clean energy, and generate a large amount of biogas slurry rich in carbon and nitrogen at the same time [3]. At present, the volume of biogas slurry produced by biogas projects in China has exceeded 1 billion tons each year [4,5]. Biogas slurry may cause serious pollution to the soil and groundwater if not disposed of in time, due to the high organic content and poor biodegradability [6]. The application of biogas slurry to farmland is seasonal, while the biogas slurry produced by biogas projects is uninterrupted. Meanwhile, the current land carrying capacity around biogas projects in China is limited. In addition, the transportation of a huge yield of biogas slurry to remote farmland will increase the cost, and it is difficult to fundamentally solve the problem [7]. Therefore, choosing an effective way to reasonably deal with biogas slurry is the only way to promote the sustainable development of biogas projects.

Common treatment technologies for biogas slurry mainly include the direct return to farmland, oxidation pond treatment, biochemical treatment, and membrane treatment [8,9,10]. However, both the return to farmland directly and the treatment of oxidation ponds require a large amount of land [11,12]. Meanwhile, biochemical treatment (such as sequencing batch activated sludge method and contact oxidation method) cannot realize the resource utilization of nutrients in biogas slurry. Membrane treatment can effectively reduce the volume of biogas slurry, recycle clean water, and greatly increase the nutrient content of biogas slurry [13]. However, there is serious membrane fouling during the membrane concentration process due to the characteristics of biogas slurry components [14]. Thus, the selection of membrane treatment technology with a low fouling tendency is of great significance for the efficient treatment of biogas slurry and the reduction of operating costs.

Conventional membrane technologies such as ultrafiltration membrane (UF), nanofiltration membrane (NF), and reverse osmosis membrane (RO) have been widely used in various fields such as industrial water treatment and municipal sewage treatment [15,16,17]. Traditional membrane separation technologies such as UF, NF, and RO are driven by external pressure. In contrast, forward osmosis (FO), an osmosis-driven membrane process, has been proposed as a low-stain alternative for treating challenging waste streams [18,19,20]. FO membranes have high selectivity, low fouling tendency, high fouling reversibility, and consume little energy when properly treating wastewater [21]. Therefore, the technology of FO membrane has a good application prospect in the field of biogas slurry concentration.

Optimizing the operating parameters can improve the filtration efficiency of the entire FO membrane system, thereby controlling membrane fouling and prolonging the service life of the membrane. The operating parameters that affect the concentration of the FO membrane mainly include the type of the draw solution, the cross-flow velocity, and the temperature. Since other factors are limited by equipment conditions, it is very important to select the appropriate type of draw solution for the concentration of biogas slurry by FO membrane treatment. At the same time, there are few studies on the membrane fouling mechanism during the FO membrane treatment of biogas slurry. Identifying membrane fouling substances through membrane characterization technology and revealing the mechanism of membrane fouling can provide a theoretical basis for further FO membrane treatment [10]. Thus, further research is needed to verify the effects of different draw solutions on FO membranes in biogas slurry treatment and the related membrane fouling characteristics.

In this study, the performance of different types of draw solution on the FO membrane concentration process of biogas slurry was compared, and the type of draw solution that was most suitable for FO membrane treatment was screened. Meanwhile, the main substances affecting the fouling of the FO membrane were identified through the membrane characterization, and the membrane fouling mechanism of the FO membrane concentration process for biogas slurry was explored. Results from this study will provide unique insights into the development of FO membranes for resource recovery from agricultural wastewater.

## 2. Materials and Methods

### 2.1. Experiment Materials

Biogas slurry was collected from a post-fermentation temporary storage tank in a local commercial pig farm (Qingdao, China). The collected biogas slurry is the supernatant after natural sedimentation in the temporary storage tank. Basic physicochemical properties of the biogas slurry were measured in triplicate according to the analytical methods described in Section 2.3. Specifically, the biogas slurry contained 4549 ± 324 mg/L chemical oxygen demand (COD), 38.5 ± 2.1 mg/L total phosphorus (TP), 943 ± 23 mg/L total nitrogen (TN), 564 ± 32 mg/L NH_4_^+^. The pH and electrical conductivity of the biogas slurry were 7.26 ± 0.08 and 8.46 ± 0.24 mS/cm, respectively.

A conventional polyamide membrane from Hydration Technology Innovations (HTI, Albany) was used in this study. The HTI membrane consisted of a thin selective polyamide active layer on top of a porous polysulfone supporting layer [22].

### 2.2. Experimental Protocol

**Figure 1:**
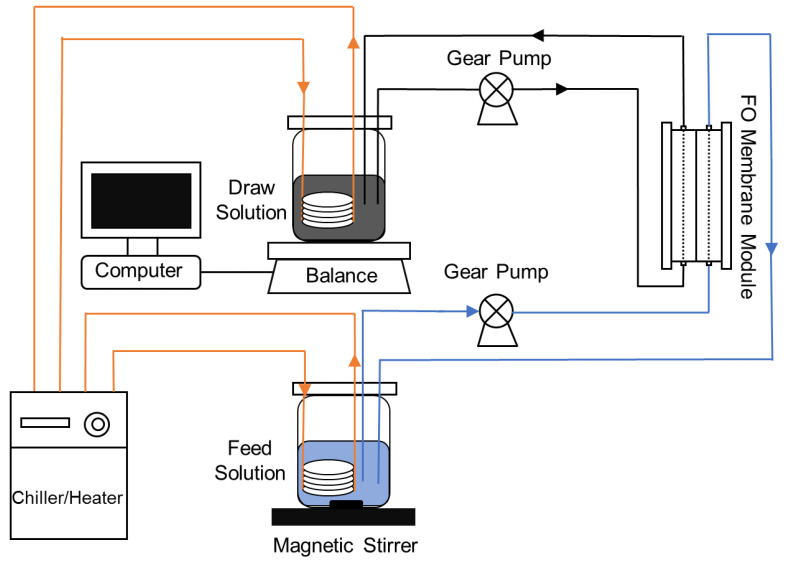
Schematic diagram of the bench-scale FO system.

A bench-scale FO system with a cross-flow membrane cell and two variable speed gear pumps was employed (Figure 1). The membrane cell consisted of two separated acrylic blocks to hold a flat-sheet membrane without any physical support. The draw solution reservoir was placed on a digital balance, which was connected to a computer to automatically record weight changes for the calculation of permeate water flux. The FO system was operated in osmotic dilution mode with biogas slurry as feed solution while 0.5 mol/L sodium chloride (NaCl), magnesium chloride (MgCl_2_), and sodium acetate (CH_3_COONa) as the draw solution, respectively. The initial volumes of the feed solution and draw solution were both 500 mL. The membrane active layer faces the feed solution. Each experiment ended when the observed water flux decreased to a negligible level. Samples (5 mL) were extracted from the feed solution at intervals during the FO operation. The most suitable draw solution was screened out according to the water recovery rate, and the membrane fouling of this treatment was analyzed.

### 2.3. Analytic Methods

Key water quality parameters of biogas slurry samples were measured according to standard methods. The specific method is the same as the previous article [22]. In addition, atomic force microscopy (AFM, Nanoman, Billerica, MA, USA) was used to observe the three-dimensional morphology of the membrane surface. The morphology and composition characteristics of the membrane surface were analyzed by scanning electron microscope combined with an energy dispersive spectrometer (JCM-6000, JEOL, Tokyo, Japan). Membrane surface functional groups were characterized by attenuated total reflection-fourier transform infrared (ATR-FTIR) spectroscopy (IRAffinity-1, Shimadzu, Kyoto, Japan) [23].

## 3. Results and Discussion

### 3.1. Water Flux and Water Recovery Rate

**Figure 2:**
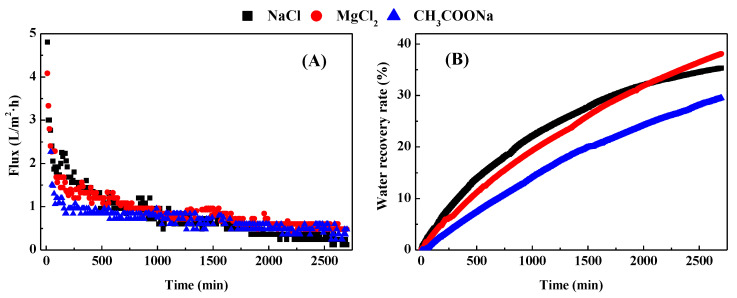
Water fluxes of each treatment (**A**) and their water recovery rate (**B**) during concentration of biogas slurry. NaCl represents the treatment with 0.5 mol/L NaCl as the draw solution. MgCl_2_ represents the treatment with 0.5 mol/L MgCl_2_ as the draw solution. CH_3_COONa represents the treatment with 0.5 mol/L CH_3_COONa as the draw solution. The FO process was operated in the osmotic dilution mode with 500 mL biogas slurry and 500 mL different draw solution as the initial feed and draw solutions at a cross-flow velocity of 8.3 cm/s, respectively.

There was a continuous decrease in water flux for each treatment during the FO membrane concentration of biogas slurry (Figure 2A). The decrease in water flux can be attributed to membrane fouling, osmotic dilution of the draw solution, and concentration changes in the feed solution. Either a concentrated feed solution or a diluted draw solution can reduce the osmotic driving force, thereby reducing the water flux [18]. NaCl as the draw solution produced the highest initial water flux, followed by MgCl_2_ and then CH_3_COONa. This result may be due to the different diffusion coefficients of these draw solutions [24]. Meanwhile, considerable flux drops were observed at the beginning of all three experiments. Membrane fouling appears to play an important role in this case due to the limited initial feed concentration. Compared with other treatments, the water flux of the treatment with NaCl as the draw solution decreased more greatly. The reason may be that the fouling layer formed rapidly on the membrane surface increased the membrane resistance due to the high initial water flux in this treatment. In addition, although the initial flux of NaCl treatment was more than 15% higher than others, the flux of each treatment decreased to 0.24–0.36 L/m^2^·h after 40 h. The water recovery rate of each treatment increased rapidly in the initial stage of membrane treatment during the initial water flux of each treatment was higher. With the reduction of the flux of each treatment, the increase in the water recovery rate tends to be flat. The MgCl_2_ treatment could recover the most water (water recovery rate was 38.1%) from the biogas slurry, while the CH_3_COONa treatment had the lowest water recovery rate of 29.5% during the period when the water flux dropped to a negligible level (Figure 2B). The water recovery rate of NaCl treatment was the highest at 2050 min before the membrane concentration operation, but the water recovery rate gradually became stable with the formation of membrane fouling. Furthermore, the surface area or active site of the membrane may also be a potential factor affecting flux and water recovery.

### 3.2. Basic Characteristics, Organic and Nutrient Enrichment

**Figure 3:**
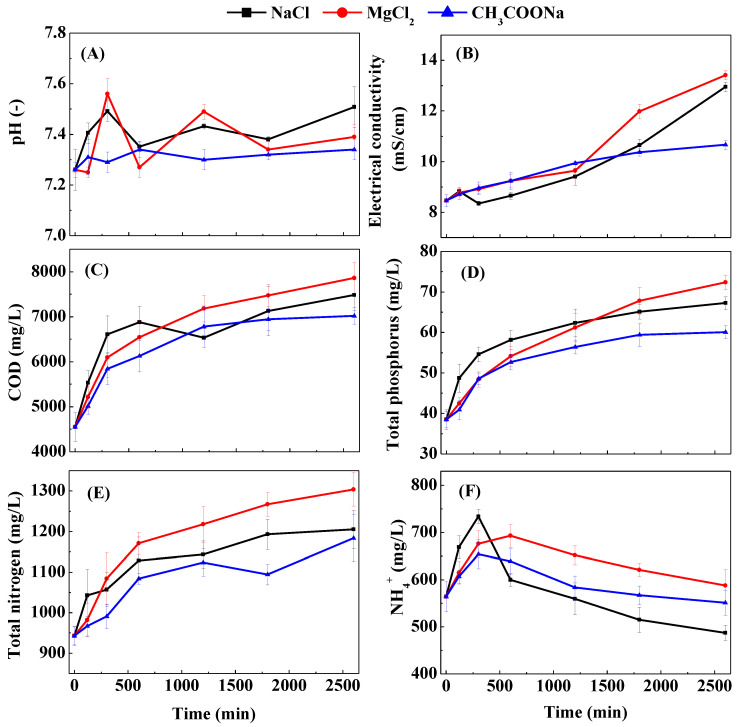
Change in the basic characteristics: pH (**A**), electrical conductivity (**B**), COD (**C**), total phosphorus (**D**), total nitrogen (**E**), NH_4_^+^ (**F**) of each treatment. Error bar represents standard deviation from duplicate experiments.

There was a certain increase in pH in the fluctuations for each treatment (Figure 3A). This trend can be attributed to the diffusion of protons between the feed and draw solutions to maintain electroneutrality on both sides of the membrane [25]. High pH can adversely affect microbial viability and membrane performance [26]. In this study, the pH rise in the feed solution was all less than 5% when the water flux was reduced to a lower level for each treatment. This phenomenon also proves that the reverse salt flux of the three extracts used in this study is lower at this concentration. The electrical conductivity of each treatment increased significantly as the FO membrane concentration progressed (Figure 3B). On the one hand, this increasing trend is due to an increase in the salt ion content as the feed solution is concentrated. On the other hand, the reverse osmosis of some solutes in the draw solution into the feed may also play a role. The electrical conductivity value of CH_3_COONa treatment increased by about 26.1% when the water flux decreased to a lower level, which was significantly lower than others by more than 50%. Considering that the water flux of other treatments was significantly higher than the CH_3_COONa treatment, the increase in salt ion content due to the concentrated feed solution should be the main reason for the increase in electrical conductivity in this study.

The organic matter and nutrients were greatly enriched while the biogas slurry was concentrated in the FO membrane with different draw solutions (Figure 3C–F). When the water flux of each treatment was reduced to a lower level, the COD content in the feed solution was concentrated by more than 1.5 times (Figure 3C). There is no difference in the rejection of organic matter since each treatment uses the same type of FO membrane [27,28]. Therefore, the lower COD concentration of CH_3_COONa treated as the draw solution was mainly attributed to its lower water recovery rate (Figure 2B). The TP content increased in a consistent trend for each FO membrane treatment as the concentration progressed (Figure 3D). Previous studies have shown that the FO membrane can almost completely retain phosphorus ions due to their large hydration radius and electrostatic repulsion to the negatively charged membrane surface [29]. The concentration of TP content in the feed solution of the MgCl_2_ treatment was the best (1.9 times) compared with the other treatments when the water flux was reduced to a lower level, which was due to the higher water recovery rate when the concentration experiment was completed.

Compared with organics and phosphorus, nitrogen was much less concentrated by the FO membranes of each treatment (Figure 3E,F). The TN content in the biogas slurry was concentrated about 1.2–1.4 times using different draw solutions. This result may be due to the low rejection of nitrogen (especially NH_4_^+^) by the FO membrane. In this study, NH_4_^+^ accounted for 40–65% of TN in biogas slurry. Previous studies have shown that the total nitrogen in biogas slurry is mainly caused by NH_4_^+^ due to the absence of nitrification in the ammoniation of organic matter and anaerobic digestion [7,30]. At the same time, NH_4_^+^ will be converted into NO_2_^−^ or NO_3_^−^ in the FO concentration process, and these nitrogen species are retained by the FO membrane to a certain extent [31,32]. In addition, the low accumulation of TN can also be attributed to its volatilization and/or attachment to the membrane surface during the concentration process, which may be due to changes in the pH of the feed solution [22]. A previous study also reported that TN volatilization exceeded 15% during the process of concentrating fecal water by reverse osmosis membranes [33,34]. Meanwhile, microbial activity also contributes to the consumption of TN [26].

The NH_4_^+^ content in the biogas slurry was not significantly concentrated by different draw solution treatments (Figure 3F). This phenomenon may be attributed to the small radius (0.104 nm) and electrostatic attraction of NH_4_^+^ [35,36]. Meanwhile, NH_4_^+^ can be converted into NH_3_, which is also one of the reasons for the low concentration ratio of NH_4_^+^ [37]. In addition, previous investigators have suggested that the reduction in NH_4_^+^ content in the feed solution may also be partly attributable to struvite precipitation due to increased concentrations of related ions [38]. The treatment of MgCl_2_ treatment had a weak concentration effect on NH_4_^+^ compared with other treatments, which was affected by the higher recovery rate. However, the NH_4_^+^ content of the NaCl treatment was the lowest after the completion of FO concentration, which was about 0.86 times the initial value. This result may be due to the high water flux in the early stage of the treatment. Kedwell (2018) observed that the loss of ammonia was exacerbated by the increase in the water flux during the FO concentration of the digested sludge concentrate [37].

### 3.3. Membrane Surface Topography

**Figure 4:**
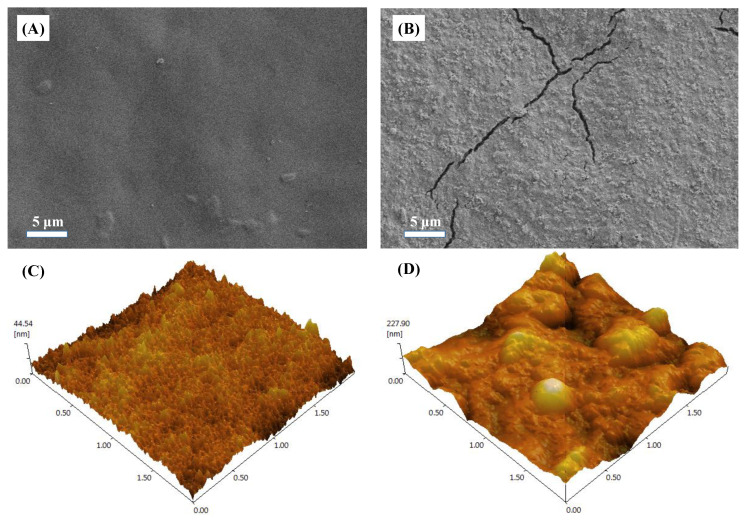
SEM and AFM measurements of the active layer of the pristine membrane (**A**,**C**) and fouled membrane (**B**,**D**) after concentrating biogas slurry. The fouled membrane is taken from 0.5 mol/L MgCl_2_ as the drawing solution to process the fouled membrane after the membrane concentration is completed.

A filter cake layer composed of macromolecular particles was formed on the membrane surface after concentration compared with the initial state of the FO membrane (Figure 4A,B). The membrane fouling layer has the characteristics of a rough surface and obvious large particles. This fouling layer structure is better than the gel layer fouling in terms of filtration resistance and permeability characteristics [39]. Stereoscopic images of the membrane surface measured by atomic force microscopy (AFM) showed increased roughness after membrane fouling (Figure 4C,D). The initial surface roughness of the FO membrane was about 44 nm, and the surface roughness increased by about five times after membrane fouling. The increased surface roughness of the FO membrane will strengthen the adhesion of organic matter and microorganisms, resulting in more serious membrane fouling [40].

### 3.4. Membrane Surface Element Distribution

**Figure 5:**
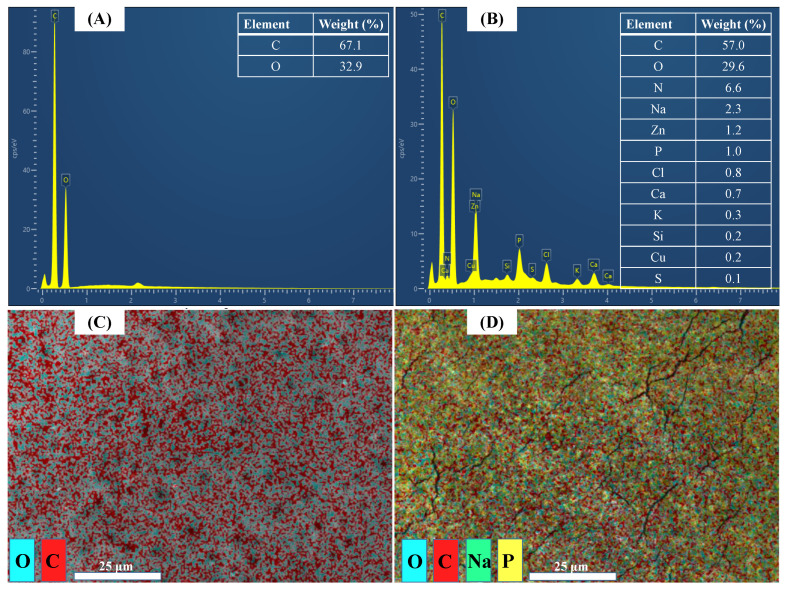
Point and face scan for EDS measurements of the active layer of the pristine membrane (**A**,**C**) and fouled membrane (**B**,**D**) after concentrating biogas slurry. The fouled membrane is denoted in Figure 4.

The stained cake layer has been covered with the original FO membrane (Figure 5A,B). Compared with the initial FO membrane, in addition to the organic substances mainly composed of C and O elements in the fouled layer (which can also be proved by the analysis in Section 3.5), inorganic elements such as Na, Zn, and Ca were also detected. The results prove that the membrane fouling in the FO concentration process of biogas slurry is caused by both organic and inorganic pollutants. Ivnitsky et al. (2007) also found that the interaction of organic matter and inorganic matter is the main cause of membrane fouling in the research of using membrane technology to treat municipal wastewater [41]. The C element accounts for 57% of the main components of the cake layer, indicating that organic pollution is still the main pollution in membrane fouling. Moreover, the Mg element was not detected in a large amount, though the draw solution was MgCl_2_ and partially passed through the FO membrane into the feed solution. This may be due to the low content of Mg in the feed solution, which is not precipitated and adhered to the membrane surface with the concentration of the biogas slurry. Therefore, it is inferred that most of the Cl elements in the membrane fouling layer come from the precipitation and deposition after the biogas slurry is concentrated. It shows that the FO membrane can effectively block the movement of the draw solution to the feed solution side in the process of biogas slurry membrane concentration.

Surface scan energy spectrogram analysis proved once again that C and O elements were uniformly distributed on the initial FO membrane surface (Figure 5C), while the membrane fouling cake layer completely covered the membrane surface (Figure 5D). The C and P elements were distributed in stripes on the membrane surface in the membrane fouling cake layer, which may be due to the uneven deposition caused by the influence of the water flow characteristics. The results of previous studies also showed that the precipitates tended to be precipitated near the water outlet because the water flow rate was lower and the concentration was higher [42].

### 3.5. Membrane Surface Functional Groups

**Figure 6:**
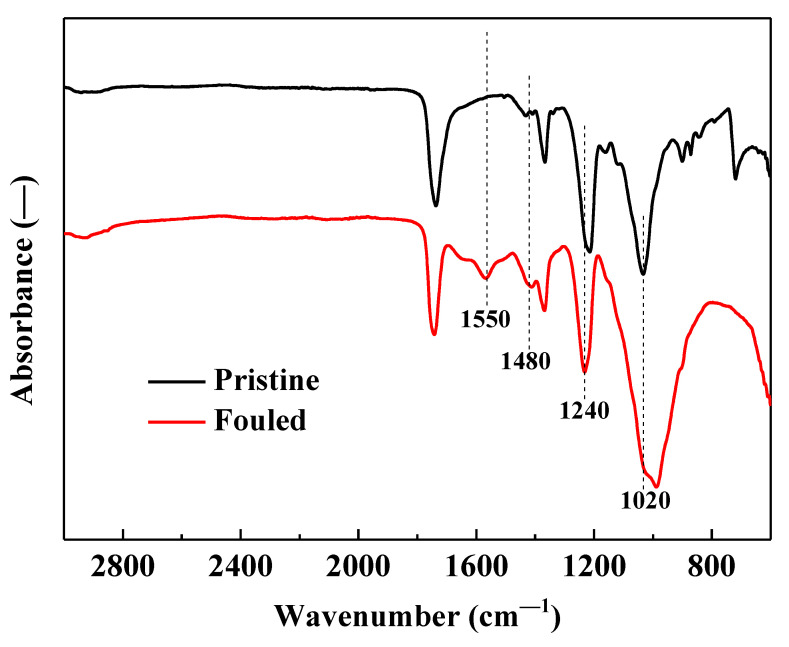
FTIR measurements of the active layer of pristine membrane and fouled membrane after concentrating biogas slurry. The fouled membrane is denoted in Figure 4.

The absorption band of the membrane surface after membrane fouling was different from that of the original FO membrane (Figure 6). The spectrum around 700–900 cm^−1^ tends to become flat after membrane fouling, indicating that the membrane concentration process has played a certain role in the change of the microstructure of the membrane surface. The main reason for this phenomenon in this study is that the membrane fouling layer shields part of the FO membrane surface. The characteristic peak around 1550 cm^−1^ indicates the existence of olefin (C=C) or amide I aliphatic structure (C=O) and amide II (C–N–H) bond on the membrane surface after fouling. Previous studies have shown that amide II mainly exists in proteins [43]. Proteins are an important component of membrane fouling. Previous studies have shown that small organics such as proteins can pass through FO membranes [44]. Considering that there is no characteristic peak at this absorption band in the original FO membrane, it shows that the adsorption on the membrane surface can have an important impact on the concentration efficiency during the membrane concentration process. Different characteristic peaks were detected at 1240 and 1480 cm^−1^ in the FO membrane before and after fouling, reflecting the changes of unsaturated pollutants such as humic substances after membrane fouling. Meanwhile, it can also be observed from the spectrum that the FO membrane has a large absorption peak near 1020 cm^−1^, which becomes no longer obvious after fouling. In fact, the characteristic peak here mainly reflects the presence of C–O, which indicates that the polysaccharides produced during the concentration of biogas slurry are less deposited on the membrane surface [45]. Combining the inorganic elements indicated by the membrane fouling layer (Figure 5B) in Section 3.5, it shows that the membrane fouling during the biogas slurry FO membrane concentration process is caused by both organic and inorganic fouling, and the fouling layer is scattered on the surface of the FO membrane (Figure 5D). Similar results were also reported by Soler-Cabezas et al. during the membrane treatment process of digested sludge midstream [46].

## 4. Conclusions

The results show that FO membrane treatment can realize the concentration of biogas slurry. MgCl_2_ as the draw solution has the best effect on the concentration of biogas slurry, and the specific performance is that the water recovery rate is 7.9% and 22.6% higher than that of NaCl and CH_3_COONa, respectively. The different draw solutions all contributed to the efficient retention of most organics (concentrated by more than 1.5 times) and TP (concentrated about 1.6–1.9 times), resulting in their substantial enrichment in the feed solution. In contrast, each treatment was ineffective at retaining nitrogen (concentrated about 1.2–1.4 times), resulting in lower TN and NH_4_^+^ rejection. The cake layer that appeared after the biogas slurry was concentrated covered the surface of the FO membrane. Some functional groups were detected on the surface after membrane fouling, such as C–O and C=C. Moreover, the C element accounts for 57% of the main components of the cake layer after the membrane fouling. Membrane fouling is caused by both organic fouling and inorganic fouling, of which organic fouling is the main reason.

## Data Availability

The datasets analyzed during the current study are available from the corresponding author on reasonable request.
